# Mutations in the DNA-Binding Domain of NR2E3 Affect *In Vivo* Dimerization and Interaction with CRX

**DOI:** 10.1371/journal.pone.0007379

**Published:** 2009-10-12

**Authors:** Raphael Roduit, Pascal Escher, Daniel F. Schorderet

**Affiliations:** 1 IRO, Institute for Research in Ophthalmology, Sion, Switzerland; 2 Department of Ophthalmology, University of Lausanne, Lausanne, Switzerland; 3 EPFL, Ecole Polytechnique Fédérale de Lausanne, Lausanne, Switzerland; INSERM U862, France

## Abstract

**Background:**

NR2E3 (PNR) is an orphan nuclear receptor essential for proper photoreceptor determination and differentiation. In humans, mutations in NR2E3 have been associated with the recessively inherited enhanced short wavelength sensitive (S-) cone syndrome (ESCS) and, more recently, with autosomal dominant retinitis pigmentosa (adRP). NR2E3 acts as a suppressor of the cone generation program in late mitotic retinal progenitor cells. In adult rod photoreceptors, NR2E3 represses cone-specific gene expression and acts in concert with the transcription factors CRX and NRL to activate rod-specific genes. NR2E3 and CRX have been shown to physically interact *in vitro* through their respective DNA-binding domains (DBD). The DBD also contributes to homo- and heterodimerization of nuclear receptors.

**Methodology/Principal Findings:**

We analyzed NR2E3 homodimerization and NR2E3/CRX complex formation in an *in vivo* situation by Bioluminescence Resonance Energy Transfer (BRET^2^). NR2E3 wild-type protein formed homodimers in transiently transfected HEK293T cells. NR2E3 homodimerization was impaired in presence of disease-causing mutations in the DBD, except for the p.R76Q and p.R104W mutant proteins. Strikingly, the adRP-linked p.G56R mutant protein interacted with CRX with a similar efficiency to that of NR2E3 wild-type and p.R311Q proteins. In contrast, all other NR2E3 DBD-mutant proteins did not interact with CRX. The p.G56R mutant protein was also more effective in abolishing the potentiation of *rhodospin* gene transactivation by the NR2E3 wild-type protein. In addition, the p.G56R mutant enhanced the transrepression of the *M-* and *S-opsin* promoter, while all other NR2E3 DBD-mutants did not.

**Conclusions/Significance:**

These results suggest different disease mechanisms in adRP- and ESCS-patients carrying NR2E3 mutations. Titration of CRX by the p.G56R mutant protein acting as a repressor in *trans* may account for the severe clinical phenotype in adRP patients.

## Introduction

NR2E3 (MIM#604485) is a photoreceptor-specific nuclear receptor (PNR) that belongs to the nuclear hormone receptor superfamily of ligand-modulated transcription factors [1]. The physiological function of NR2E3 is to regulate the proper differentiation and maturation of rod and cone photoreceptors, in an intricate regulatory network including the cone-rod homeobox (CRX) (MIM#602225) and the neural retina leucine zipper (NRL) (MIM#162080) transcription factors [2]. In late mitotic retinal progenitor cells, NR2E3 is thought to suppress the cone generation program [3], while in adult differentiated rods, NR2E3 exerts a dual function by repressing cone-specific genes [4], [5] and by activating several rod-specific genes, including rhodopsin [4], [6], [7].

All nuclear receptors (NRs) share a common structural organization composed of four main domains [8]. First, the highly variable N-terminal A/B domain comprises a ligand-independent activation function (AF-1). Second, the most conserved C domain forms a 70-amino acid long DNA-binding domain (DBD) consisting of two Cys_4_ zinc fingers. The C-terminus of the first Cys_4_ zinc finger comprises the so-called P-box that specifically contacts nucleotides located in the DNA major groove. Three discriminatory amino acids (underlined below) determine DNA binding, EGCKS and EGCKG in NR2 family members and orphan NRs [1]. NR2E subfamily members exhibit exceptional P-boxes with NGCSG sequence in NR2E3 and DGCSG in NR2E1 and NR2E2. The consensus core motif recognized by NR2E proteins appears to be AAGTCA [1], [9]. The region between the first two Cys residues of the second Cys_4_ zinc finger forms the so-called D-box, which is involved in DBD dimerization and recognition of the spacing between two adjacent core motifs. Third, the flexible D domain, or so-called hinge domain, links the DBD to the ligand-binding domain (LBD) and contains the nuclear localization signal which may overlap on the DBD. Fourth, the C-terminal E/F domain, or LBD, consists of a conserved secondary structure formed by 12 α-helixes, containing a strong dimerization function and the ligand-dependent AF-2 transactivation function.

The first NRs most likely acted as monomers in a ligand-independent fashion [10]. NRs then acquired the ability to homo- or heterodimerize and to interact with specific ligands, allowing the regulation of more diverse and more complex physiological processes. Within the NR2E subfamily, Drosophila tailless (NR2E2) and its chick and mouse orthologs Tlx (NR2E1) bind their target genes as monomers [11], [12]. Homodimers have also been reported to bind DNA, but to a lesser extent. Interestingly, NR2E3 also acts as a transcriptional repressor (see above), but, in contrast to NR2E1, *in vitro* DNA binding experiments suggested binding to a direct repeat of the core motif spaced by one nucleotide (so-called DR1 response element), the consensus binding site being 5′-(A/G)AG(A/G)TCAAA(A/G)(A/G)TCA-3′ [1], [5]. It is therefore tempting to speculate that in addition to unique P-box and D-box sequences and to a spatial expression uniquely restricted to photoreceptors, NR2E3 dimer formation can increase target specificity when compared to NR2E1, another NR expressed in the retina [12].

In humans, mutations in NR2E3 have first been associated with the recessively inherited enhanced short wavelength sensitive (S-) cone syndrome (ESCS) (MIM #268100) [13]. ESCS is characterized by unique full-field and spectral electroretinographic findings with hyperfunction of S-cones (‘blue’ cones) and impaired M-cones, L-cones and rods function [14]. To date, 32 different mutations located in the evolutionary-conserved DBD and LBD of NR2E3 have been linked to ESCS with c.932G>A (p.R311Q) being the most prevalent [13], [15]–[19]. A c.166G>A (p.G56R) mutation located in the first Cys_4_ zinc finger of NR2E3 gene was shown to cause autosomal dominant (ad) retinitis pigmentosa (RP) (adRP), termed RP37 (MIM #611131) [20]–[22]. The phenotype corresponded to that seen in classic adRP, with progressive degeneration of rods and subsequent involvement of cones.

In this study, we analysed by Bioluminescence Resonance Energy Transfer 2 (BRET^2^) [23]–[27], NR2E3 homodimerization and interaction with CRX for all reported NR2E3 mutations located in the DBD. Transcriptional activity of the different mutants was also tested on *rhodopsin*, S- and *M-opsin* promoter reporter constructs. These analyses showed a distinct *in vivo* protein-protein interaction of the adRP-linked p.G56R mutant protein with CRX, comparable to that of wild-type NR2E3, but contrasting with the other ESCS-linked DBD mutants. This provided a novel molecular basis for the clinical differences seen in human patients affected by adRP versus those affected by ESCS [19].

## Results

### NR2E3 homodimerization in HEK293T cells

**Figure 1:**
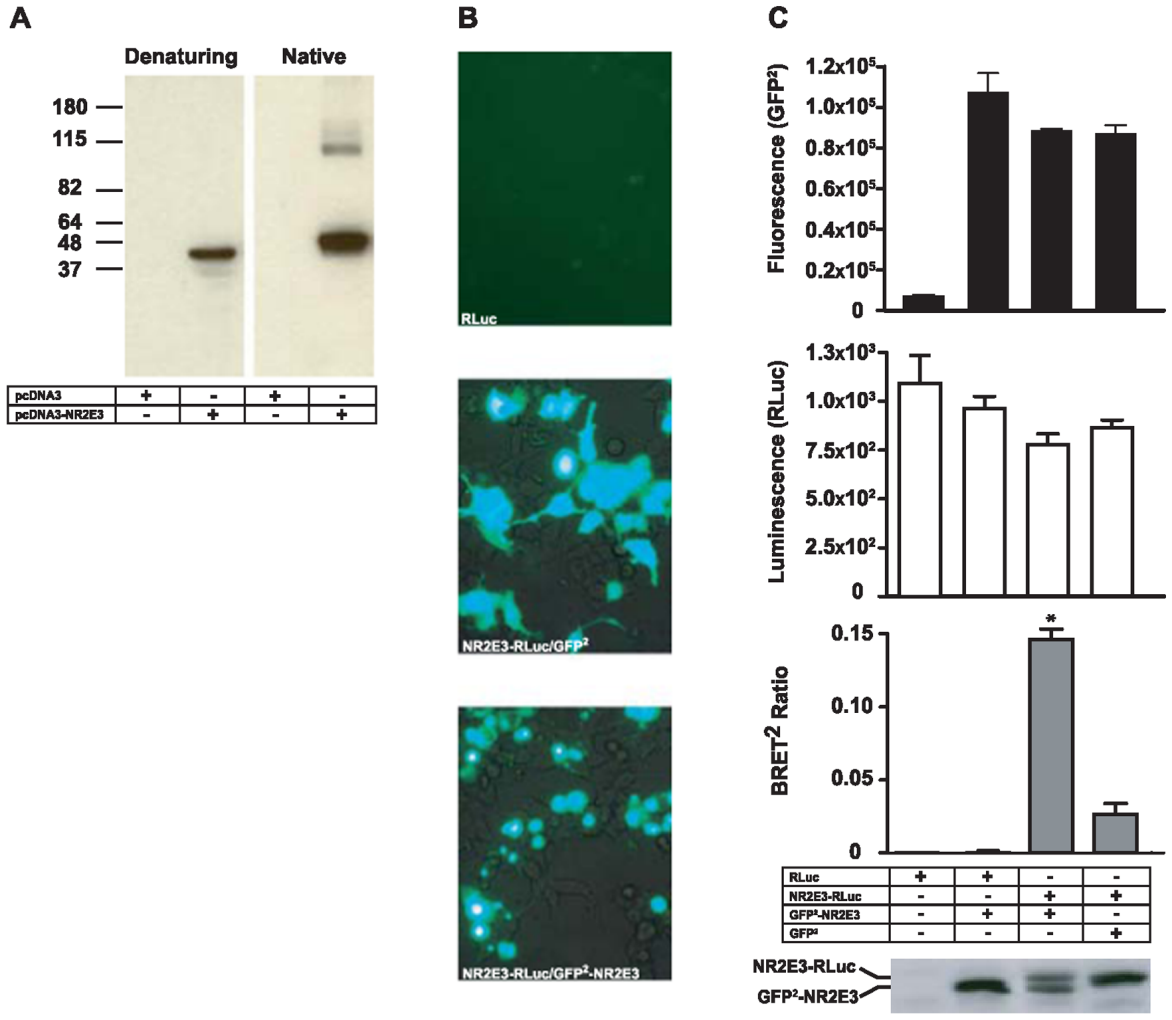
NR2E3 homodimerization analyzed by Western blot and Bioluminescence Resonance Energy Transfer (BRET^2^). A) HEK293T cells were transiently transfected in 60-mm plates with either 3 µg of an empty pcDNA3 vector or a pcDNA-NR2E3-His/C vector. Forty-eight hours after transfection, cell lysates were separated on a 8% PAA-gel in both non-denaturing (left panel) and denaturing (right panel) conditions. NR2E3 was immunodetected by Western blot with an anti-NR2E3 antibody. Molecular weights are indicated on the left side in kDa. B–C) For BRET^2^ analysis, HEK293T cells were transiently transfected with 3 µg of RLuc, RLuc/GFP^2^-NR2E3, GFP^2^-NR2E3/NR2E3-RLuc and NR2E3-RLuc/GFP^2^ vectors. B) GFP fluorescence and localization analysis by fluorescence microscopy in cells transfected with RLuc, NR2E3-RLuc/GFP^2^ and NR2E3-RLuc/GFP^2^-NR2E3. C) NR2E3 homodimerization analyzed by BRET^2^ 48 h after transfection. GFP^2^ fluorescence was quantified at 515 nm after an excitation at 485 nm (upper graph in black bars). After addition of the renilla luciferase substrate, Coelanterazine h luciferase luminescence was measured (middle graph in white bars). Fluorescence and luminescence levels are in arbitrary units. Finally, BRET^2^ ratio was measured in the four different conditions (lower graph in grey bars). BRET^2^ sesults were expressed as mean±SEM of 3 experiments, p<0.003 (NR2E3-RLuc/GFP^2^-NR2E3) *vs.* both controls (NR2E3-RLuc/GFP^2^ and RLuc/GFP^2^-NR2E3). Protein expression of NR2E3 fused either with GFP^2^ (GFP-NR2E3: 74 kDa) or RLuc (NR2E3-RLuc: 82 kDa) was assessed by Western blot analysis. We used a 10% PAA-gel to separate proteins and an anti-NR2E3 antibody to detect NR2E3 fusion proteins (panel, below).

To analyze a potential dimerization of NR2E3, we expressed the human NR2E3 wild-type protein by transient transfection in heterologous HEK293T cells. Total protein extracts were separated by SDS-PAGE under denaturing and non-denaturing conditions (figure 1A). Western blot analysis with an anti-NR2E3 antibody detected a band of the expected size at 45 kDa under denaturing conditions, while a fainter additional reactive band was observed at 90 kDa under native conditions. This result suggested that NR2E3 was able to form homodimers *in vivo*.

To analyze the dimerization of NR2E3 *in vivo*, we resorted to the BRET^2^ technique. We transiently transfected HEK293T cells with vectors expressing the Renilla Luciferase (RLuc) fused to the C-terminus of NR2E3 (NR2E3-RLuc) and the Green Fluorescent Protein (GFP^2^) fused to the N-terminus of NR2E3 (GFP^2^-NR2E3). As negative controls, cells were transfected with vectors expressing 1) RLuc alone, 2) GFP^2^ and NR2E3-RLuc and 3) RLuc and GFP^2^-NR2E3. Figure 1B showed the both negative controls 1 and 2, and positive experiment. Moreover, this fluorescence imaging showed that the GFP^2^-NR2E3 fusion protein was correctly localized to the nucleus, whereas the GFP^2^ protein showed both cytosolic and nuclear expression. By BRET^2^ analysis, we were able to measure comparable GFP^2^ fluorescence levels in cells expressing either the GFP^2^ or the GFP^2^–NR2E3 proteins, whereas low fluorescence levels were observed in controls transfected only with the RLuc expression vector (figure 1C, upper panel). In addition, when the Renilla Luciferase substrate, coelanterazine h, was added to the cells, we observed similar levels of luminescence in all experimental conditions (figure 1C, middle panel). These data confirmed that similar levels of protein expression occurred in both control and experimental conditions. Finally, we measured the BRET^2^ ratio (see Material & Methods) and observed a significant increase only in the presence of both GFP^2^-NR2E3 and NR2E3-RLuc (figure 1C, lower panel). In controls, where either GFP^2^-NR2E3 was expressed with RLuc, or NR2E3-RLuc with GFP^2^, the BRET^2^ ratios were very low.

**Figure 2:**
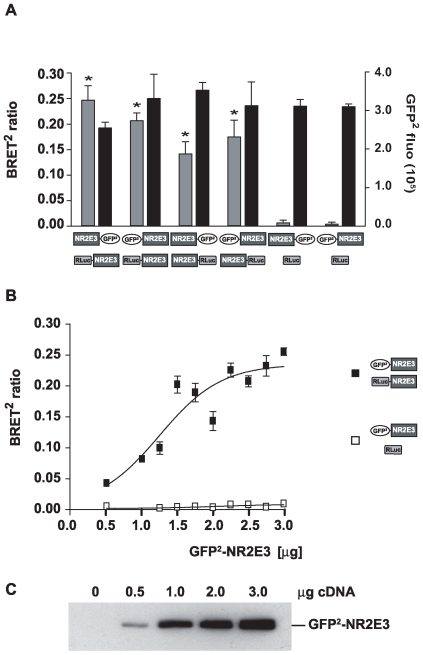
NR2E3 homodimerization is orientation-independent. A) HEK293T cells were transiently transfected with 3 µg of NR2E3-GFP^2^, GFP^2^-NR2E3, NR2E3-RLuc and RLuc-NR2E3 vectors, as indicated. Control transfections were performed with renilla alone (RLuc) and NR2E3 fused to GFP^2^ (NR2E3-GFP^2^ and GFP^2^-NR2E3). BRET^2^ ratio (grey column) and GFP^2^ fluorescence (black column) were measured and sesults were expressed as mean±SEM of 3 experiments, p<0.003 *vs.* controls. B) HEK293T cells were transiently transfected with 3 µg of RLuc or NR2E3-RLuc vectors, to keep the expression level of both proteins stable in each condition. At the same time, cells were transfected with increasing concentrations (0.5 µg to 3 µg) of GFP^2^-NR2E3 vector. BRET^2^ ratio was measured and sesults were expressed as mean±SEM of 4 experiments. (see also supplemental figure 1A). C) Protein expression of various GFP^2^-NR2E3 concentrations was assessed by Western blot analysis using an anti-GFP antibody.

We then tested whether NR2E3 dimer formation was dependent upon the localization of the donor (GFP^2^) or the acceptor (RLuc) protein in the NR2E3 fusion protein. We transfected HEK293T cells with vectors expressing NR2E3 fused to the N-terminus or the C-terminus of either GFP^2^ or RLuc. Again, a significant increase in BRET^2^ ratios was observed in presence of all combinations of NR2E3 fusion proteins, while protein expression levels (NR2E3-GFP^2^ or GFP^2^-NR2E3 fluorescence) were comparable in each condition. These results suggested that the orientation of the fusions was not interfering with dimerization (figure 2A).

To exclude the possibility that the increase in BRET^2^ ratios was due to non-specific interactions of overexpressed proteins, we performed a dose-dependency experiment (figures 2B, 2C and S1A). A constant amount of RLuc or RLuc-NR2E3 was expressed in the presence of increasing amounts of GFP^2^-NR2E3. In these conditions, we observed a dose-dependent increase of the BRET^2^ ratios, with a significant increase even at the lowest GFP^2^-NR2E3 expression levels (0.5 µg of expression vector). This further confirmed the specificity of the NR2E3 homodimerization.

### NR2E3/CRX interactions in HEK293T cells

**Figure 3:**
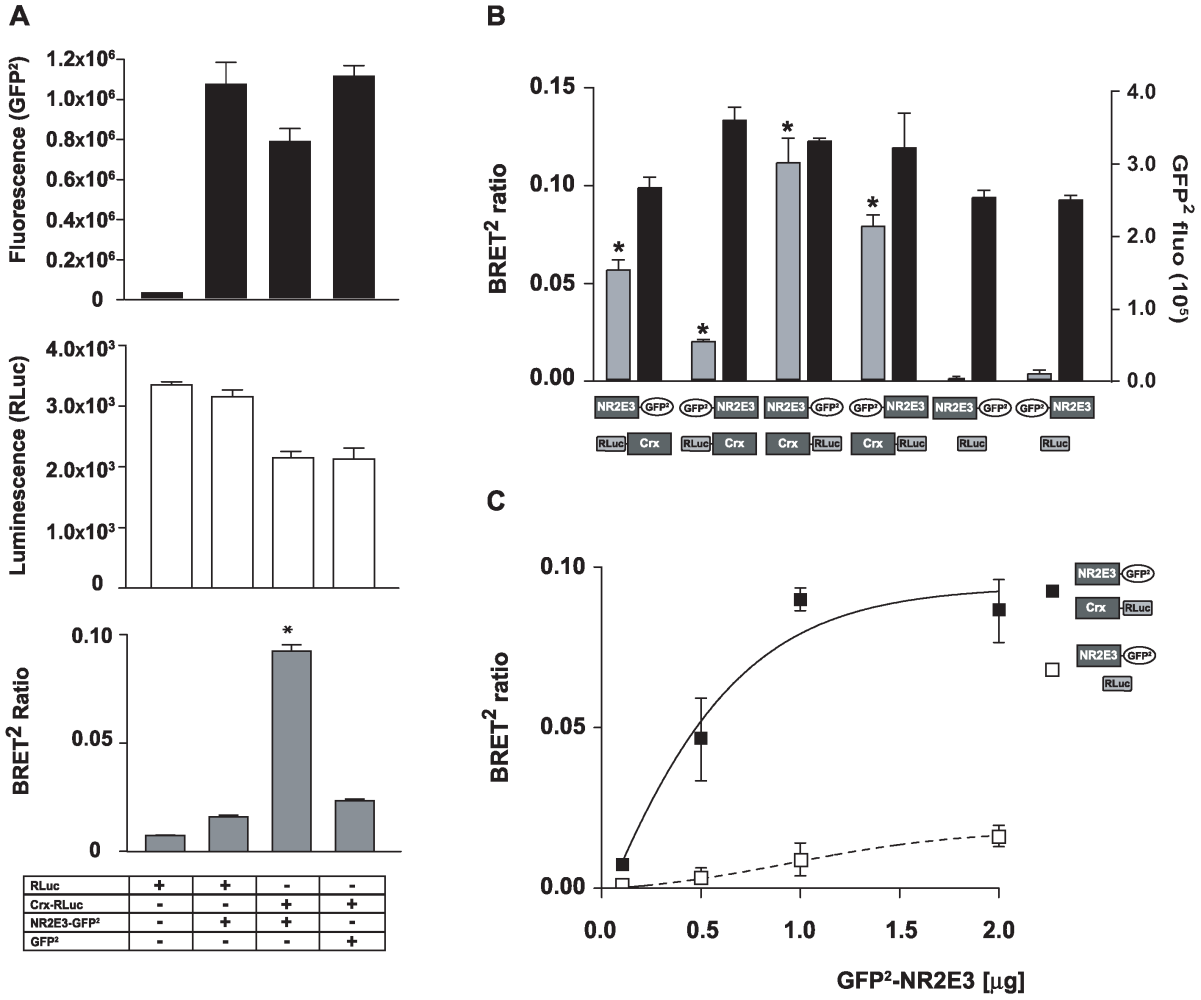
NR2E3 interacts with CRX. A) HEK293T cells were transiently transfected with 3 µg of RLuc, RLuc/GFP^2^-NR2E3, GFP^2^-NR2E3/Crx-RLuc and Crx-RLuc/GFP^2^ vectors. BRET^2^ analysis was performed 48 h after transfection. First we quantified the GFP^2^ fluorescence (at 515 nm) after an excitation at 485 nm (upper graph in black). Then, we added the renilla luciferase substrate, Coelanterazine h, to the cell and measured the luciferase luminescence (middle graph in white). Finally, BRET^2^ ratio was measured in the four different conditions (lower graph in grey). B) HEK293T cells were transiently transfected with 3 µg of NR2E3-GFP^2^, GFP^2^-NR2E3, Crx-RLuc and RLuc-Crx vectors, as indicated. Control transfections were performed with renilla alone (RLuc) and NR2E3 fused to GFP^2^ (NR2E3-GFP^2^ and GFP^2^-NR2E3). BRET^2^ results (grey column) and GFP^2^ fluorescence (black column) were expressed as mean±SEM of 2 experiments. *p<0.0001 *vs.* Ctrl. C) We transfected HEK293T cells with 3 µg of RLuc or Crx-RLuc vectors to keep the expression level of both proteins stable in each condition. At the same time, we transfected cells with increasing concentrations (0.5 µg to 2 µg) of NR2E3-GFP^2^ vector. BRET^2^ ratio was measured and sesults were expressed as mean±SEM of 3 experiments, *p<0.0001 *vs.* Ctrl (see also supplemental figure 1B).

As a physical interaction between NR2E3 and CRX through their respective DBDs was previous reported in vitro [4], we decided to analyze this interaction by BRET^2^ techniques. We transiently transfected HEK293T cells with vectors expressing GFP^2^ or NR2E3-GFP^2^, in presence of either RLuc or a fusion protein where the human CRX protein was fused in N-terminus of RLuc (Crx-RLuc). We were able to measure similar GFP^2^ fluorescence levels in cells expressing either GFP^2^ or NR2E3-GFP^2^, while low fluorescence levels were observed in control conditions with RLuc (figure 3A, upper panel). We observed a slight decrease in Renilla luminescence levels in presence of Crx-RLuc, as compared to RLuc (figure 3A, middle panel). Importantly, we observed a significant increase of the BRET^2^ ratio only in presence of both NR2E3-GFP^2^ and Crx-RLuc (figure 3A, lower panel). BRET^2^ ratios were lower in both control conditions (RLuc/NR2E3-GFP^2^ or Crx-RLuc/GFP^2^).

We then tested whether the localization of CRX in the fusion protein was important for NR2E3/CRX protein interactions (figure 3B). The highest BRET^2^ ratios, i.e. the most efficient NR2E3/CRX protein interactions, were obtained when CRX was located in the C-terminus of the renilla (RLuc-Crx), while this ratio was significantly decreased when CRX was fused to the N-terminus of RLuc (Crx-RLuc). Similar protein expression levels (NR2E3-GFP^2^ or GFP^2^-NR2E3 fluorescence) were observed in all performed conditions. These results showed that the orientation of the fusion proteins was important and suggested that the N-terminal part of CRX, where the DBD is located, had to be accessible for efficient NR2E3/CRX interactions.

We also performed a dose-dependency experiment, in presence of a constant amount of either RLuc or Crx-RLuc on the one hand, and, increasing amounts of NR2E3-GFP^2^ on the other hand (figures 3C and S1B). In this experiment, we observed a significant increase of the BRET^2^ ratio even at low amounts of NR2E3-GFP^2^ (0.5 µg). Taken together, these results confirmed a specific *in vivo* interaction between NR2E3 and CRX.

### BRET^2^ analysis of DBD mutations on NR2E3 dimerization and CRX interaction

To elucidate the molecular mechanisms by which mutations located in a same functional domain of NR2E3, i.e. the DBD, cause different clinical phenotypes, we chose to generate eight NR2E3 mutant proteins and tested their effect on dimerization. Seven of these mutations were localized in the DBD, i.e. the adRP-linked p.G56R mutation and the ESCS-linked p.R76Q, p.R76W, p.G88W, p.R97H, p.R104Q and p.R104W mutations. Additionally, we tested the common causal mutation for ESCS located in the LBD, p.R311Q. All mutations were introduced in the pcDNA3.1/HisC-hNR2E3 mammalian expression vector by site-directed mutagenesis (Table S1) and then subcloned into GFP^2^ vectors. The different NR2E3 mutant proteins (GFP^2^-NR2E3_MUT_) showed proper nuclear localization and protein expression levels as tested in transiently transfected HEK293T cells (figures S2 and S3A).

**Figure 4:**
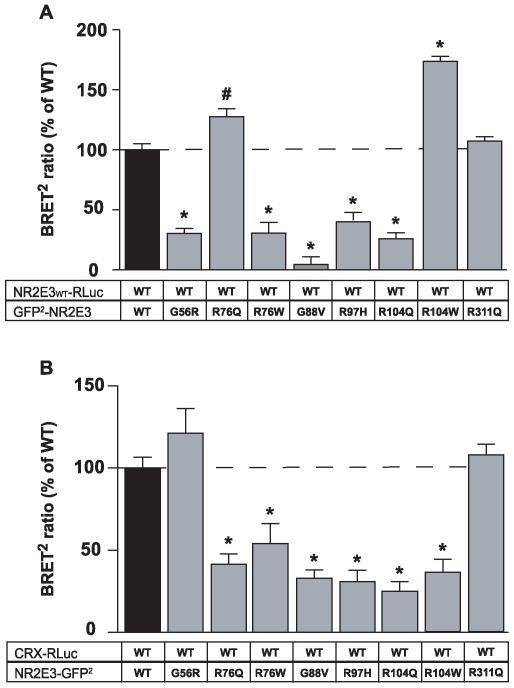
Role of NR2E3 DBD mutations in the dimerization process and in the interaction with CRX. A) HEK293T cells were transiently transfected with 2 µg of vectors expressing the wild-type (WT) NR2E3 fused to renilla luciferase (NR2E3_WT_-RLuc) and with 4 µg vector expressing mutants of NR2E3 fused to the GFP^2^ (GFP^2^-NR2E3_MUT_). Control condition with RLuc alone and GFP^2^-NR2E3_WT_ served to calculated the background BRET^2^ ratio. Luminescence ratio was measured as described in material and methods, and sesults were expressed as % of GFP^2^-NR2E3_WT_/NR2E3_WT_-RLuc and as mean±SEM of 3 experiments, * p<0.0001 and # p<0.01. (see supplemental figure 4 for BRET^2^ titration curve). B) HEK293T cells were transiently transfected with 2 µg of vectors expressing the CRX fused to renilla luciferase (Crx-RLuc) and with 4 µg of vector expressing mutants of NR2E3 fused to the GFP^2^ (NR2E3_MUT_-GFP^2^). Control condition with RLuc alone and GFP^2^-NR2E3_WT_ served to calculated the background BRET^2^ ratio. Luminescence ratio was measured as described in material and methods, and sesults were expressed as % of NR2E3_WT_-GFP^2^/Crx-RLuc and as mean±SEM of 2 experiments, *p<0.003. (see supplemental figure 5 for BRET^2^ titration curve).

To test whether these mutations altered the homodimerization of NR2E3, we expressed wild-type NR2E3 fused to RLuc (NR2E3_WT_-RLuc) in presence of equal amounts of each NR2E3 mutant fused to GFP^2^ (GFP^2^-NR2E3_MUT_) (figure 4A). For each experiment, we performed three control transfections: 1) RLuc alone to measure background fluorescence levels, 2) GFP^2^-NR2E3 and RLuc, and 3) NR2E3-RLuc and GFP^2^ as negative controls (figure S3A). Additionally, we performed a BRET^2^ titration curve for each NR2E3 mutant fused with GFP^2^ in presence of wild-type NR2E3 fused to RLuc or with RLuc alone as negative control (figure S4). The NR2E3 mutations differentially altered NR2E3 dimerization. In comparison to the NR2E3 wild-type protein, the BRET^2^ ratio was decreased by up to 70% in presence of the p.G56R, p.R76W, p.G88V, p.R97H and p.R104Q mutant proteins, thus indicating an impaired dimer formation. Interestingly, the p.R76Q mutation increased significantly dimerization (127.8±6.6%), whereas the p.R76W mutation dramatically decreased it (30.5±9.0%). In contrast, the p.R104Q mutation decreased dimerization (25.8±5.2%), whereas the p.R104W mutation increased it strongly (173.5±4.5%). The p.R311Q mutation located in the LBD did not alter dimerization, when compared to the wild-type NR2E3 protein.

Next, we resorted to the same experimental approach to test whether CRX/NR2E3 protein interactions were altered in presence of mutations in the NR2E3 DBD. We expressed Crx-RLuc in presence of an equal amount of each NR2E3 mutant fused to GFP^2^ (NR2E3_MUT_-GFP^2^) (figure 4B). For negative controls, cells were transfected with: 1) RLuc alone to measure background fluorescence levels, 2) GFP^2^-NR2E3 and RLuc, and 3) CRX-RLuc and GFP^2^ (figure S3B). Additionally, we performed a BRET^2^ titration curve for each NR2E3 mutant fused to GFP^2^ in presence of wild-type CRX fused to RLuc or with RLuc alone as negative control (figure S5). All ESCS-linked mutants, except p.R311Q, showed a significant decrease of interaction with CRX (50% or more). Remarkably, the CRX interaction of the adRP-linked p.G56R mutant was comparable to that of the NR2E3 wild-type protein.

### Mutations in the NR2E3 DBD abolish DNA binding

We examined whether NR2E3 mutations affected DNA-binding. The pcDNA3.1/HisC-hNR2E3 wildtype and mutant plasmids were *in vitro* transcribed/translated in reticulocyte lysates and tested for DNA-binding by electrophoretic mobility-shift assay (EMSA) on the reported synthetic DR1 containing two AAGTCA half-sites [1]. No DNA-binding was observed for all seven DBD-mutants, whereas the p.R311Q LBD-mutant was able to bind the DNA response element (figure S6).

### Impaired transcriptional activity of NR2E3 DBD mutant proteins

We then tested the effect of the NR2E3 mutant proteins on CRX/NRL-mediated transactivation by transient transfection assays in HEK293T cells. CRX and NRL synergistically activate *rhodopsin*, *S-* and *M-opsin* gene expression, and NR2E3 further potentiates CRX/NRL-mediated transactivation of *rhodopsin* expression by about 3-fold, but represses CRX/NRL-mediated transactivation of *S-* and *M-opsin* expression [4], [7], [21].

**Figure 5:**
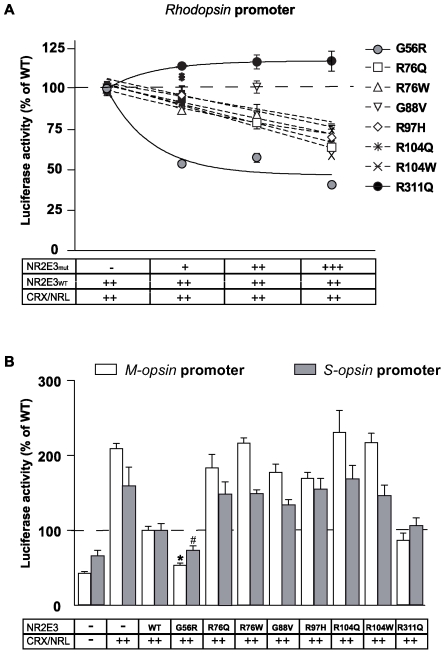
Differential transcriptional activity of NR2E3 DBD mutants. HEK293T cells were transiently transfected with *rhodopsin* (A) or *S-* and *M-opsin* (B) luciferase reporter constructs. A) NR2E3 wild-type (NR2E3_WT_), CRX and NRL expression vectors (30 ng each) were present in all wells. Transcriptional activity was tested in presence of increasing amounts (+:15 ng; ++:30 ng; +++:45 ng) of NR2E3-DBD mutants proteins (NR2E3_m_: p.G56R, p.R76Q, p.R76W, p.G88W, p.R97H, p.R104Q, p.R104W and p.R311Q). Four to six independent experiments were performed in duplicate in 12-well plates using the luciferase assay system (Promega). Luciferase activity was normalized to β-galactosidase activity. Normalized luciferase activities of cells transfected with NR2E3_WT_, CRX and NRL were set to 100%. Error bars represent S.E.M. Regression curves were performed for each NR2E3-DBD mutant and ANOVA tests were performed (see Results section). B) NR2E3 wild-type (WT) or above-mentioned mutants, CRX and NRL expression vectors (30 ng each) were present in all wells except the negative control (−) representing the background luciferase activity. Normalized luciferase activities of cells transfected with NR2E3_WT_, CRX and NRL were set to 100% and sesults were expressed as means±SEM of 4–6 experiments, *p<0.0001 and #p<0.03 for p.G56R *vs.* WT.

NR2E3-dependent potentiation of CRX/NRL-mediated transactivation of a bovine *rhodopsin* promoter fragment (BR225-Luc) was set to 100% for each experiment, and, by transfecting increasing amounts of NR2E3 mutant proteins, we tested their effect on *rhodopsin* transactivation (figure 5A). The ratios of NR2E3_WT_:NR2E3_MUT_ were 2∶1, 2∶2 and 2∶3. NR2E3 mutant activity could be separated in three different groups, depending on the transactivation effect. First, the adRP-linked p.G56R mutant showed a highly significant decrease in NR2E3_WT_-mediated *rhodopsin* transactivation by 50% already at a NR2E3_WT_/NR2E3_G56R_ ratio of 2∶1 (p<0.01 by ANOVA test for p.G56R *vs* all other mutants). Higher NR2E3_WT_/NR2E3_G56R_ ratios (2∶2 and 2∶3) did not further decrease *rhodopsin* promoter activity. Second, all six ESCS-linked DBD-mutants significantly repressed NR2E3_WT_-mediated *rhodopsin* transactivation, but to a lesser extent. Except for p.R104Q, this repression was significant at a NR2E3_WT_/NR2E3_MUT_ ratio of 2∶1 (p<0.05 by ANOVA of all ESCS-linked DBD mutants *vs* p.R311Q). At a NR2E3_WT_/NR2E3_MUT_ ratio of 2∶3, repression was significant for all DBD mutants (p<0.01 by ANOVA of all ESCS-linked DBD mutants *vs* p.R311Q,). Third, the LBD-mutation p.R311Q did not alter wild-type NR2E3 transactivation at any NR2E3_WT_/NR2E3_R311Q_ ratio.

With respect to repression of CRX/NRL-mediated transactivation of *S-* and *M-opsin* gene expression, the different NR2E3 mutations segregated into three different groups (figure 5B). First, the adRP-linked p.G56R mutant repressed both promoter fragment more efficiently than the NR2E3 wild-type protein, the resulting *S-* and *M-opsin* promoter activity being only about 50% of the wild-type one (p<0.0001 by ANOVA *vs* other mutants) [21]. Second, the ESCS-linked DBD-mutants did not significantly repress *S-* and *M-opsin* promoter transactivation, when compared to NR2E3 wild-type (p<0.01 by ANOVA). Third, the LBD-mutant p.R311Q repressed *S-* and *M-opsin* promoter activity similarly to wild-type NR2E3.

### Homology modeling of the NR2E3 homodimer DNA-binding complex

**Figure 6:**
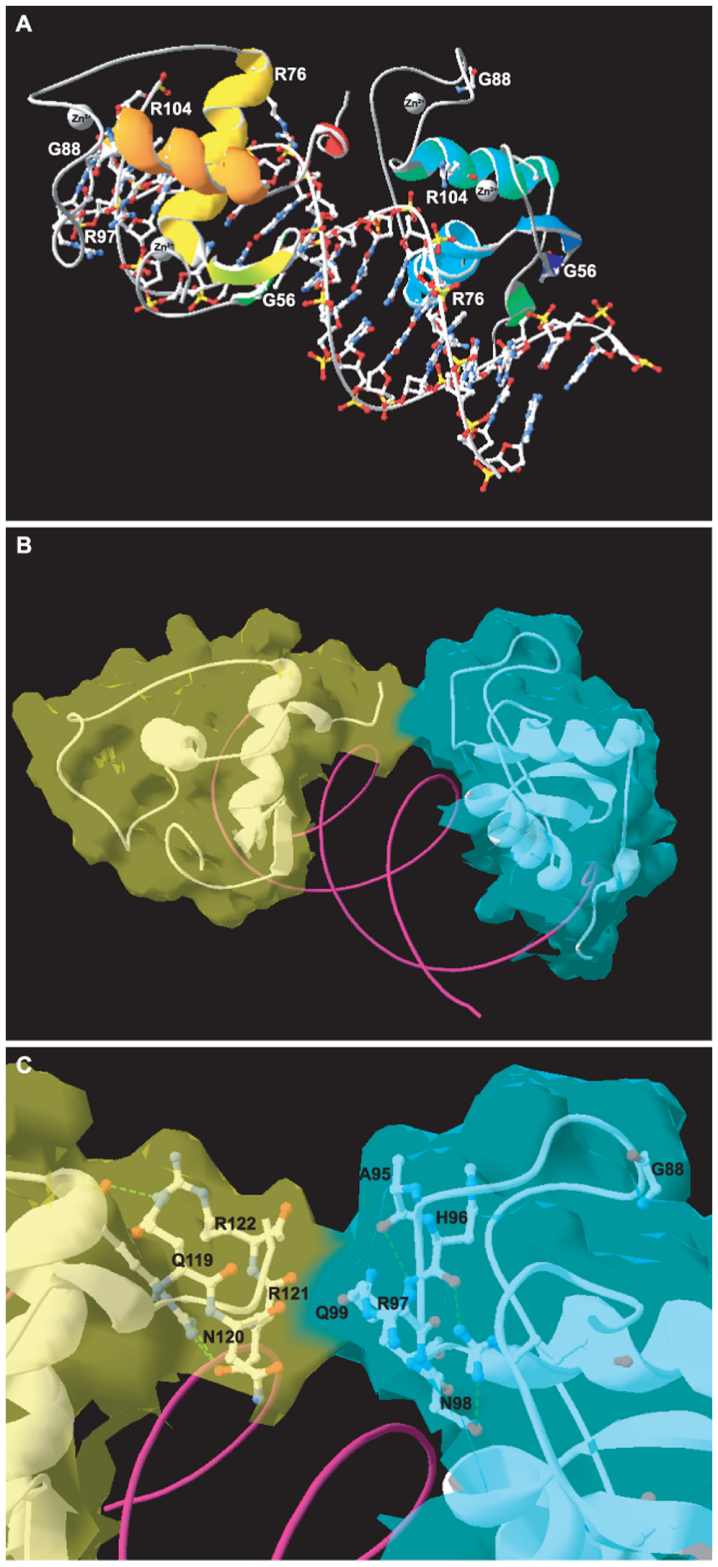
Homology modeling of the NR2E3 homodimer DNA-binding complex. A) Ribbon view of the NR2E3 DBD homodimer bound to a DR1. Secondary structures of the NR2E3 DBD are shown as ribbons, *i.e.* starting from the N-terminus, 2 β-sheets of the first Cys_4_ Zinc finger, and then the 3 α-helixes extending to the C-terminus (see supplemental Figure 4). The left NR2E3 DBD monomer is shown in yellow-orange, and the right one in blue-green colours. Residues mutated in patients are indicated and shown with sidechains. For the DNA double helix all side chains are shown. Zinc ions are shown as grey spheres. Residue G56 is located in the β-sheets of the first Cys_4_ Zinc finger and spatially segregated from the other analyzed mutations. B) Molecular surface view of the NR2E3 homodimer DNA-binding complex. Molecular surface were represented in light-green for the left monomer and in light-blue for the right monomer. The α-helix of the T/A box of the left NR2E3 monomer (light green) interacts with the second Cys_4_ Zinc finger of the right NR2E3 monomer (light-blue). The DNA double helix is shown only as ribbon and colored in magenta. C) Close-up view of the dimerization interface. Residues involved in protein interactions are indicated and shown with sidechains. Figures were made with DeepView 4.0 program.

To integrate the obtained functional data into related structural information, we performed homology modeling of the NR2E3 homodimer bound to a DR1 DNA sequence (figure 6). According to crystallographic data obtained for RXR/RXR and RXR/RAR dimers [28], [29], the NR2E3 homodimer bound to a DR1 is thought to interact through the α-helix of the T/A box of the 5′-monomer and the second Cys_4_-Zinc finger of the 3′-monomer. This model allowed us to evaluate potential functions of the residues that are mutated in patients. Residues R76, R97 and R104 are predicted to directly interact with DNA (figure 6A, S7 and S8). Residues R97 is, in addition, located in the second Cys_4_-Zinc finger forming the dimerization interface, whereas residue G88 is located in a loop close by (figure 6C). Notably, residue G56 is located in the β-sheets of the first Cys_4_-Zinc finger and spatially segregated from the other analyzed mutations (figure 6A).

## Discussion

In the present work, we analyzed by BRET^2^ the effect of disease-causing mutations located in the DBD of NR2E3. For all NR2E3 mutants we performed a BRET^2^ titration curve with a constant concentration of donor fluorochrome and an increased concentration of acceptor fluorochrome. This approach used in previous BRET studies [26], [30], [31] provides strong support to our proposed mechanism leading to ESCS or adRP syndromes.

Like for all NRs, mutations in the DBD of NR2E3 abolished DNA-binding, as assessed by *in vitro* binding assays (figure S6). Absence of DNA-binding is therefore insufficient to explain the variety of clinical phenotypes observed in patients carrying mutations in this domain (reviewed in [19]). For instance, the adRP phenotype present in patients carrying the p.G56R mutation cannot be explained by a defect in DNA-binding alone [20]–[22]. Because NR2E3 had been shown to interact with CRX [4], we hypothesized that the transcriptional activity of CRX could be affected by structural changes present in NR2E3 mutant proteins. We therefore evaluated by BRET^2^ analysis whether NR2E3 DBD mutations affected the interaction with CRX causing phenotypic variations among patients. Remarkably, the adRP-linked p.G56R mutant protein interacted with CRX as did the wild-type protein (figure 4B and S5). This was in sharp contrast to all other ESCS-linked NR2E3 DBD mutant proteins, *i.e.* p.R76Q, p.R76W, p.G88V, p.R97H, p.R104Q and p.R104W. Consistent with this unique CRX interaction, the p.G56R mutant protein repressed in HEK293T-based transient transactivation assays the cone-specific *S-opsin* and *M-opsin* promoters, down to levels where CRX/NRL-mediated transactivation was affected [21]. All ESCS-linked NR2E3 DBD mutants not interacting with CRX, *i.e.* p.R76Q, p.R76W, p.G88V, p.R97H, p.104Q and p.104W, had similar transcriptional activities on *rhodopsin*, *S-* and *M-opsin* promoters (figures 4 and 5). This was somewhat different from a previous report where p.R76W and p.R97H mutant proteins differentially interacted *in vitro* with CRX as assessed by co-immunoprecipitation, but exerted the same effects on *M-opsin* and *rhodopsin* transactivation [4].

**Figure 7:**
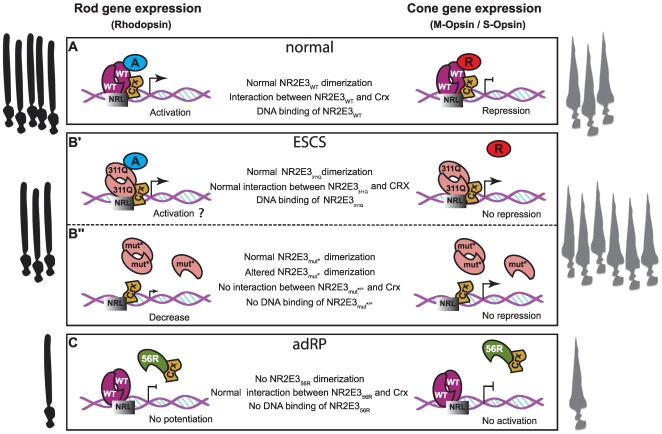
Hypotheses about transcriptional activities of NR2E3. A) In the “normal” situation, wild-type NR2E3 activates rod-specific genes (*rhodopsin* promoter) and represses cone-specific genes (*M-opsin and S-opsin* promoter) in concert with CRX and NRL. B') In ESCS patients, rhodopsin expression appears not to be affected in the presence of the p.R311Q LBD mutant protein. The molecular mechanism is unknown, but may involve posttranslational modifications or differential binding to cofactors. However, repression of cone genes is released, because interaction of the p.R311Q mutant protein with its co-repressor is impaired [4]. B″) In ESCS patients carrying mutations in the DBD, in the absence of DNA binding, NR2E3 does not potentiate CRX/NRL-mediated *rhodopsin* transactivation and does not repress cone-specific gene expression. C) In adRP patients, the p.G56R mutant protein acts as a repressor in *trans* of CRX-mediated photoreceptor-specfic gene expression. WT: wild type; 311Q: p.R311Q; mut*: p.R76Q, p.R104W; mut°: p.R76W, p.G88V, p.R97H, p.R104Q; 56R: p.G56R.

The data presented here provide a novel molecular basis underlying adRP caused by the p.G56R mutant protein (figure 7). In this model the p.G56R mutant protein binds to CRX, hinders DNA-binding of the CRX/NR2E3-p.G56R heterodimer and acts as a repressor in *trans*. Because CRX is a master regulator of photoreceptor development, titrating active CRX protein levels is expected to have more profound effects than would the dominant negative activity towards NR2E3 wild type protein. In support of impaired CRX activity is not only the severe clinical phenotype of adRP, but also the mode of inheritance, i.e. one copy of the disease allele is sufficient to cause p.G56R-linked adRP, whereas the recessively inherited ESCS patients are either homozygous or compound heterozygous carriers of NR2E3 mutations.

NR2E3/CRX interactions analyzed by BRET^2^ also showed the importance of a freely accessible N-terminal DBD of CRX to interact with NR2E3. Indeed, we observed a low energy transfer when the Renilla luciferase was fused to the N-terminal part of CRX (RLuc-Crx), in contrast to the Crx-RLuc fusion protein, where high energy transfer was observed (figure 3B). We cannot exclude that low BRET^2^ signals were due to conformational changes that affect the distance between the donor and the acceptor, but our results are consistent with previous co-immunoprecipitation and yeast two-hybrid assays, showing that CRX interacts with NR2E3 through its N-terminal DBD region [4].

In addition to abolishing DNA-binding and modulating interaction with CRX (see above), modulation of NR2E3 homodimerization by mutations in the DBD could be an additional factor affecting *in vivo* NR2E3 activity. We therefore used BRET^2^ to investigate the dimerization potential of NR2E3 wild-type and mutant proteins. BRET^2^ provided the first *in vivo* evidence of NR2E3 homodimerization in transiently transfected HEK293T cells (figures 1 and 2). We did observe NR2E3 dimer formation in non-denaturing gel electrophoresis (figure 1A), but the signal corresponding to a NR2E3 dimer was weak with respect to the signal of the monomer. The influence of gel electrophoresis conditions on the oligomerization potential of another NR2 family member, the retinoid X receptor, had been studied previously in detail [32] and might underlie our observations.

The p.G56R mutant protein showed impaired dimerization with NR2E3 wild type protein as analyzed by BRET^2^ (figure 4A and S4). This result was in contradiction with our previous *in vitro* data where the p.G56R mutant protein dimerized with NR2E3 wild-type protein in EMSA analysis [21]. The new BRET^2^ data obtained in living cells is not in support of the previously suggested dominant negative activity of the p.G56R mutant protein, but the distinct interaction with CRX is consistent with the sesults previously obtained in transactivation assays on *rhodopsin*, *M-* and *S-opsin* promoter constructs. BRET^2^ analysis performed in living cells made it therefore possible to clarify our previous *in vitro* data and to propose this new disease mechanism [21].

Consistent with the observed impaired dimerization, R97 is located in the dimerization interface (figures 4A and 6C). Interestingly, when DNA-interacting residues R76 and R104 are mutated into Q or W, opposing effects on dimerization were observed by BRET^2^. Mutant proteins p.R76W and p.R104Q showed impaired homodimerization, but not p.R76Q and p.R104W mutant proteins. Mutating R76 abolishes two hydrogen bonds with DNA and the presence of a bulky hydrophobic side chain in the p.R76W mutant protein might displace the C-terminal α ~helix of the T/A box that forms the DBD dimerization interface (figure S8). In contrast, the side chain of residue 104 is directed towards the inside of the DBD, leaving intact the dimerization interfaces. The dramatic effect of the p.G88V mutation on NR2E3-dimerization could be due to a displacement of the second Cys_4_-Zinc finger (figure 6C). However, in the absence of crystallographic data, these interpretations remain speculative.

In conclusion, BRET^2^ has proven to be a valuable and reliable method to analyze NR2E3 and CRX protein-protein interactions. This functional analysis in living cells lead to further understanding of potential disease mechanisms in *NR2E3*-linked retinal degenerations. Figure 7 summarized hypothetic mechanisms supported by our results (this study and [21]) and others studies ([4], [7]). All analyzed mutations in the DBD disrupt DNA binding, but the persistent association with CRX causes the adRP-linked p.G56R mutant protein to act as a repressor of CRX in *trans*. The clinical phenotype resulting from the other *NR2E3* mutations in the DBD are similar to those observed in presence of mutations in the LBD, i.e. ESCS. We have previously shown in heterologous transactivation assays that repression of cone-specific promoters by the LBD mutant protein p.R311Q was impaired in presence of the co-repressor atrophin [33]. The absence of NR2E3 repressor function on cone-specific promoters very likely causes ESCS, and at least three different molecular mechanisms may be involved: 1) absence of NR2E3 protein because of nonsense mutations and aberrant splicing; 2) absence of DNA binding because of mutations in the DBD; 3) impaired corepressor binding because of mutations in the LBD.

Recently, the dual function of NR2E3 in mature rods, *i.e.* repression of cone-specific genes and activation of several rod-specific genes, has been shown to be regulated by the E3 small ubiquitin-related modifier (SUMO) ligase PIAS3 (protein inhibitor of activated Stat3) [33]. PIAS3-dependent SUMOylation converts NR2E3 into a potent repressor of cone-specific genes. Whether *NR2E3* mutations occurring in patients affect SUMOylation or other posttranslational modifications and this, in turn, affects DNA binding or protein-protein interactions, remains elusive and will require further studies.

## Materials and Methods

### Expression vectors

Bioluminescence Resonance Energy Transfer (BRET^2^) vectors, encoding the humanized Renilla luciferase (hRluc) and the humanized green fluorescent protein 2 (hGFP^2^) were purchased from PerkinElmer-BioSignal (Montreal, QC, Canada). The plasmids pcDNA3.1/HisC-hNR2E3 and pcDNA3.1/HisC-hCRX expressing the human NR2E3 and CRX, respectively, were kindly provided by Shiming Chen [4]. To obtain all NR2E3 mutants we performed mutagenesis according to the QuikChange®II Site-Directed Mutagenesis Kit. Briefly, pcDNA3.1/HisC-hNR2E3 was amplified with oligos described in Table S1 using PfuUltra polymerase (Stratagene; Cedar Creek, TX). For RLuc-fusion proteins, PCR amplification (oligonucleotides available on request) of wild-type and mutants NR2E3, and CRX were subcloned, in the hRluc-C1 or -N3 vectors, respectively. We obtained the Rluc-NR2E3, NR2E3-Rluc, Rluc-Crx and Crx-Rluc vectors, which express chimeric proteins. For GFP^2^-fusion proteins, PCR amplification (oligonucleotides available on request) of wild-type and mutants NR2E3 were subcloned, in the hGFP^2^-C3 or -N3 vectors, respectively. We obtained the GFP-NR2E3 and NR2E3-GFP vectors, which express chimeric proteins. All constructs were verified by nucleotide sequencing.

### Cell Culture Condition and Transfection

Human embryonic kidney (HEK) 293T cells were cultured at 37°C in an atmosphere of 5% CO_2_, in Dulbecco's modified Eagle's medium (DMEM) supplemented with 10% FCS, 100 U/ml penicillin and 100 µg/ml streptomycin (#31330, Invitrogen AG, Basel, Switzerland). For BRET^2^ analyses, one day prior to the experiment, cells were seeded in 60-mm cell culture plates at 0.8×10^6^ cells. One day after plating, each 60-mm plate was transfected with the Calcium Phosphate method (ProFection®, Promega, Madison, WI) with 0.5 µg to 3 µg of plasmid depending of the experiment. The cells were harvested 48 h after transfection prior to Western blot and BRET^2^ analysis.

### BRET^2^ Assays and Fluorescence Imaging

Transfected HEK293T cells were washed with phosphate-buffered saline (PBS), detached with trypsin/EDTA, and then washed twice with Dulbecco's Phosphate-Buffered Saline (D-PBS, GIBCO, Invitrogen AG, Basel, Switzerland). Aliquots of 10^5^ cells were distributed in black 96-well microplates (Optiplate, PerkinElmer Life Sciences/Packard Biosciences) for fluorescence quantification. Filter sets were 485 nm for GFP^2^ excitation and 515 nm for emission. Cells expressing BRET^2^ donor alone (RLuc) were used to determine fluorescence background. Aliquots of cells expressing same levels of fluorescence were distributed in white 96-well microplates (Optiplate, PerkinElmer Life Sciences/Packard Biosciences) for luminescence assay. The luciferase substrate Coelenterazine h, DeepBlueC, (PerkinElmer/Biosignal; Montreal, QC) was added at a final concentration of 5 µM. Filter sets were 410 nm for luciferase emission and 515 nm for GFP^2^ emission. BRET^2^ ratio is defined as: [emission at 515 nm)/(emission 410 nm)] – Cf, where Cf corresponds to (emission at 515 nm)/(emission 410 nm) for the control experiment with Rluc construct expressed with the concerned protein fused to the GFP^2^. BRET^2^ ratio is expressed as raw data in experiments showing all negative controls. Emitted fluorescence and luminescence were measured using the Envision^®^ microplates reader (PerkinElmer/Biosignal, QC, Montreal). For the GFP^2^ fluorescence imaging, HEK293T cells were analyzed under microscope with excitation (λ_ex_ = 485 nm) and emission (λ_em_ = 515 nm) filters.

### Western Blot Analysis

HEK293T cells were cultured as described above. Then, they were washed in PBS, suspended in 100 µl sample buffer containing 20 mM Hepes, 0.5% Tween, phosphatase inhibitors cocktail 1 and 2 (Sigma #p2850, #p5726; St-Louis, MO) and Complete® protease inhibitors (Roche Applied Science, Rotkreuz, Switzerland). Cells were lysed with 3 successive freezing-thawing cycles. The detergent-insoluble material was pelleted by centrifugation at 15,000 rpm for 5 min at 4°C. The supernatant containing protein cell lysates (30 µg) were used for western blotting. For Western blot analysis, proteins were electrically transferred to PVDF filters and incubated with anti–NR2E3 (Chemicon; Millipore AG, Switzerland) or anti–GFP (Sigma-Aldrich; St.Louis, MO, USA). Secondary antibody, anti-rabbit-HRP (Amersham Biosciences; Otelfingen, Switzerland), was used to detect protein expression. Immune complexes were detected by chemiluminescence using LumiGLO (Amersham Biosciences, Otelfingen, Switzerland).

### Transcriptional Activity

HEK293T cells were cultured as described above. Cells were plated in 12-well plates and transfected at a confluence of 30% with the Calcium Phosphate method (ProFection®, Promega, Madison, WI). Per well, 30 ng of each of the expression vectors pcDNA3.1/HisC-hNR2E3 (wild-type, p.G56R, p.R76Q, p.R76W, p.G88W, p.R97H, p.R104Q, p.R104W and p.R311Q), pcDNA3.1/HisC-hCRX and pMT-NRL were used, together with 500 ng of the luciferase reporter constructs for *rhodopsin* promoter (BR225-Luc) [4], *M-opsin* promoter (Mop250-Luc) [4] or S_opsin promoter (opn1sw) [4]. As internal standard, 50 ng of plasmid CMVβ (Clontech, Moutain View, CA) encoding β-galactosidase was used. To keep the total transfected DNA quantity constant, appropriate quantities of pcDNA3.1/HisC empty vector was added in all experiments. Enzymatic activities were assessed with Luciferase Assay System (Promega, Madison, WI) and standard β-Gal assay.

### Homology modeling

Homology modeling was performed in project mode on the SWISS-MODEL server (http://swissmodel.expasy.org) using DeepView 4.0 program [34]–[36]. Amino acids 47–123 of human NR2E3 (SwissProt Acc. No. Q9Y5X4) spanning the DBD were modeled, using crystallographic data of COUP-TF DBD (PDB Acc. No. 2EBL), RXR homodimer on DR1 DNA sequence (PDB Acc. No. 1BY4) and RXR/RAR heterodimer on DR1 DNA sequence (PDB Acc. No. 1DSZ) as templates.

### Statistical Analysis

All results were expressed as means±SEM of the indicated number of experiments. Statistical significance was calculated with the Student's *t* test and ANOVA, using Prism 4.0.2 (GraphPad Software; La Jolla, CA).

## Supporting Information

Figure S1 BRET^2^ analysis of NR2E3 homodimerization and interaction with CRX. Donor saturation curves were obtained by measuring BRET^2^ ratio in the presence of fixed quantities of donor and increasing amounts of acceptor. In panel A, HEK293T cells were transiently transfected with 3 µg of RLuc or NR2E3-RLuc vectors, and an increased concentration (0.5 µg to 3 µg) of GFP^2^-NR2E3 vector. While in panel B, HEK293T cells were transiently transfected with 3 µg of RLuc or Crx-RLuc vectors and with an increased concentration (0.5 µg to 2 µg) of NR2E3-GFP^2^ vector. We pooled 5 to 6 different experiments and expressed the BRET^2^ ratio ([emission at 515 nm)/(emission 410 nm)] - Cf, where Cf corresponds to (emission at 515 nm)/(emission 410 nm) for the control experiment with Rluc construct expressed with the concerned protein fused to the GFP^2^) in function of (GFP/GFP°)/RLuc where GFP° correspond to the luminescence of the acceptor in cells expressing the BRET donr alone (RLuc). In both BRET^2^ experiments we reach a plateau with high acceptor concentration.
(1.03 MB EPS)

Figure S2 Nuclear localization of NR2E3 wild-type and mutant proteins. HEK293T cells were transiently transfected with GFP^2^-NR2E3 expression vectors. Fluorescence imaging under a Zeiss (Axiovert 200) inverted microscope was used to detect GFP^2^ fluorescence. Wild-type and mutant NR2E3 proteins were correctly localized to the nucleus.
(3.73 MB EPS)

Figure S3 Comparable expression levels of NR2E3 wild-type and mutant proteins in transiently transfected HEK293 cells. (A) HEK293T cells were transiently transfected with vectors expressing the wild-type NR2E3 (WT) fused to renilla luciferase (NR2E3_WT_-RLuc) and with mutants of NR2E3 fused to the GFP^2^ (GFP^2^-NR2E3_MUT_). Fluorescence of the GFP^2^ was measured to ensure an equal expression of GFP^2^ alone and GFP^2^-NR2E3_x_ chimera proteins in each condition. Results were expressed as % of GFP^2^-NR2E3_WT_/NR2E3_WT_-RLuc and as a mean±SEM of 3 experiments. (B) HEK293T cells were transiently transfected with vectors expressing the CRX fused to renilla luciferase (Crx-RLuc) and with mutants of NR2E3 fused to the GFP^2^ (NR2E3_MUT_-GFP^2^). Fluorescence of the GFP^2^ was measured to ensure an equal expression of GFP^2^ alone and GFP^2^-NR2E3_x_ chimera proteins in each condition. Results were expressed as % of GFP^2^-NR2E3_WT_/Crx-RLuc and as a mean±SEM of 2 experiments.
(1.26 MB EPS)

Figure S4 BRET^2^ titration curves of NR2E3 mutants in the NR2E3 dimerization. For each NR2E3 mutant, we transfected 293T cells with 2 Î¼g of RLuc alone (RLuc) or fused to NR2E3 (RLuc-NR2E3_WT_) and an increasing amount (0.5 to 4 Î¼g) of the NR2E3 wild-type or mutants fused to the GFP^2^ (GFP^2^-NR2E3_WT_ or GFP^2^-NR2E3_MUT_). Sigmoidal regression curves were performed for all graphs.
(1.25 MB EPS)

Figure S5 BRET^2^ titration curve of NR2E3 mutants in NR2E3/CRX interation. For each NR2E3 mutant we transfected 293T cells with 2 Î¼g of RLuc alone (RLuc) or fused to CRX (CRX-RLuc) and an increasing amount (0.5 to 4 Î¼g) of the NR2E3 wild-type or mutant fused to the GFP^2^ (NR2E3_WT_-GFP^2^ or NR2E3_MUT_-GFP^2^). Sigmoidal regression curves were performed for all graphs.
(2.03 MB EPS)

Figure S6 Mutations in the DBD impair DNA binding. NR2E3 wild-type and mutants proteins were *in vitro* transcribed/translated in reticulocyte lysates (TNT; Promega).; *In vitro* DNA-binding of NR2E3 proteins was tested on a radiolabeled consensus DR1 response element [1] with a 100-fold excess of either non-specific (−) or specific (+) competitor oligonucleotides. One Î¼l of programmed reticulocyte lysate was used per binding reaction. Transcription/translation efficiency was tested by western blot analysis (data not shown). The migration of the complex formed by binding of the NR2E3 dimer to the probe is indicated by a black arrow. DNA binding is only detected in the presence of wild-type NR2E3 and the LBD-mutant p.R311Q (low level of detection was observed for the p.R311Q mutant in this experiment, but higher exposition and published studies [21] showed clearly a DNA-binding for the p.R311Q mutant). All DBD mutants, i.e. p.G56R, p.R76Q, p.R76W, p.G88V, p.R97H, p.R104Q and p.R104W, did not bind to DNA. The radiolabeled probe was obtained by annealing oligonucleotides NR2E3REfor (5′-CCTTTAAAAGTCAAAAGTCAACTTCCAA-3′) and NR2E3RErev (5′-TTCCGTTGGAAGTTGACTTTTGACTTTT-3′). Radiolabeling by Klenow fill-in with 30 Î¼Ci of [Î±-^32^P]dATP (3000 Ci/mmol) (Hartmann Analytik, Braunschweig) and subsequent probe purification on Sephadex G-50 columns was according to manufacturers instructions (Roche, Basel, Switzerland). DNA-binding reactions were carried out in 20 Î¼l of 10 mM Tris (pH 7.5), 160 mM KCl, 1 mM dithiothreitol (DTT), 10% glycerol, 10 Î¼g of sonicated salmon sperm DNA (Roche), 2 Î¼g of poly(dI-dC) and 1 Î¼l of programmed reticulocyte lysate. After a 15-min incubation on ice, 1 ng of ^32^P-labeled probe was added, and incubations were continued for an additional 15 min at room temperature. DNA-protein complexes were separated from free probe on a native 4% polyacrylamide gel in 0.5 x Tris/borate/EDTA (TBE) buffer. Gels were dried and revealed by phosphorimaging (GE Healthcare, Piscataway, NJ).
(1.73 MB EPS)

Figure S7 Amino acid sequence alignment of human NR2E3 (PNR) and NR2B1 (RXRÎ±) DNA-binding domains (DBDs). The NR2E3 DBD (residues 47–123 of SwissProt Acc. No. Q9Y5X4) exhibits a sequence identity of 57.1% (44 residues) and a sequence similarity of 80.5% (62 residues) with the RXRÎ±DBD (residues 135–210 of SwissProt Acc. No. P19793). The main regions of the DBD, i.e. the two Cys4 Zn-fingers and the so-called T/A box, are indicated above the sequence alignment. Disease-causing mutations are indicated above the NR2E3 sequence. Given the high sequence homology, the structural data obtained for RXRÎ± were also indicated on the NR2E3 sequence [27], [28]. Residues directly interacting with DNA are highlighted in yellow. Residues directly involved in protein-protein interactions in the RXR homodimer are highlighted in blue. Residues involved in both DNA-binding and RXR dimerization are highlighted in grey. The Î±-helical regions are underlined. The Î±-helical region extending from the C-terminus of the first Cys4 Zn-finger is called recognition helix and contains the P-box responsible for DNA-binding specificity. The residues of the P-box are shown in italics. In the N-terminus of the second Cys4 Zn-finger, the residues of the D-box involved in dimerization are also shown in italics. A second Î±-helical region extends from the C-terminus of the second Cys4 Zn-finger. The third Î±-helix is a part of the so-called T/A box that extends beyond the region for which crystallographic data are available. Sequences were aligned with the CLUSTAL W 2.0 algorithm. Polar residues are shown in green, non-polar residues in red, basic residues in magenta, and acidic residues in blue. Below the sequence alignment, (*) denotes sequence identity, (:) denotes conserved substitutions, and, (.) denotes semi-conserved substitutions.
(1.00 MB EPS)

Figure S8 Mutation analysis by homology modeling of the NR2E3 homodimer DNA-binding complex. Amino acids 47–123 of human NR2E3 (SwissProt Acc. No. Q9Y5X4) were used for homology modelling on the SWISS-MODEL server with DeepView program (http://swissmodel.expasy.org) [30]–[32]. Crystallographic data of COUP-TF DBD (PDB Acc. No. 2EBL), RXR homodimer on DR1 DNA sequence (PDB Acc. No. 1BY4) and RXR/RAR heterodimer on DR1 DNA sequence (PDB Acc. No. 1DSZ) were used as templates. Secondary structures of the NR2E3 DBD monomer are shown as ribbons, i.e. starting from the N-terminus, 2 β-sheets of the first Cys4 zinc finger in green-yellow, the α-helix located in C-terminus of the first Cys4 zinc finger in yellow, the second α-helix located in C-terminus of the second Cys4 zinc finger in orange and the C-terminal α-helix of the T/A box in red (see also supplemental Figure 4). Residues p.R76 (left panels) and p.R104 (right panels) are shown with sidechains. For the DNA double helix all side chains are shown. Zinc ions are shown as small grey spheres. Residue p.R76 (left, upper panel) is located at the very C-terminus of the α-helix located in C-terminus of the first Cys4 zinc finger and directly contacts DNA through two hydrogen bonds (green dotted line). Both p.R76Q and p.R76W mutations disrupt hydrogen bond formation (left, middle and lower panels). The most favoured rotamers of the mutated residues all point towards the C-terminal a-helix of the T/A box. The bulky hydrophobic Trp sidechain might therefore impede dimerization. Residue p.104 (right, upper panel) is located in the middle of the α-helix located in C-terminus of the second Cys4 zinc finger. The sidechain points towards the inside of the protein, also in presence of the p.R104Q and p.R104W mutations (left, middle and lower panels).
(5.25 MB EPS)

Table S1 PCR primers used for NR2E3 mutagenesis.
(1.43 MB EPS)
